# Epigenetic Inheritance and Its Role in Evolutionary Biology: Re-Evaluation and New Perspectives

**DOI:** 10.3390/biology5020024

**Published:** 2016-05-25

**Authors:** Warren Burggren

**Affiliations:** Department of Biological Sciences, University of North Texas, 1155 Union Circle #305220, Denton, TX 76203-5017, USA; burggren@unt.edu; Tel.: +1-940-565-2591

**Keywords:** epigenetics, evolution, inheritance, natural selection, ecology, dynamics, climate change

## Abstract

Epigenetics increasingly occupies a pivotal position in our understanding of inheritance, natural selection and, perhaps, even evolution. A survey of the PubMed database, however, reveals that the great majority (>93%) of epigenetic papers have an intra-, rather than an inter-generational focus, primarily on mechanisms and disease. Approximately ~1% of epigenetic papers even mention the nexus of epigenetics, natural selection and evolution. Yet, when environments are dynamic (e.g., climate change effects), there may be an “epigenetic advantage” to phenotypic switching by epigenetic inheritance, rather than by gene mutation. An epigenetically-inherited trait can arise simultaneously in many individuals, as opposed to a single individual with a gene mutation. Moreover, a transient epigenetically-modified phenotype can be quickly “sunsetted”, with individuals reverting to the original phenotype. Thus, epigenetic phenotype switching is dynamic and temporary and can help bridge periods of environmental stress. Epigenetic inheritance likely contributes to evolution both directly and indirectly. While there is as yet incomplete evidence of direct permanent incorporation of a complex epigenetic phenotype into the genome, doubtlessly, the presence of epigenetic markers and the phenotypes they create (which may sort quite separately from the genotype within a population) will influence natural selection and, so, drive the collective genotype of a population.

## 1. Introduction

Epigenetics is rapidly earning its place as a major phenomenon in modern life sciences. Indeed, according to the PubMed database, nearly 50,000 papers referencing “epigenetics” have been published to date, with >19,000 published just since 2013. The areas of the life sciences where epigenetics is influencing disciplinary thought are widespread, but not necessarily evenly spread, between the various disciplines and taxa. Indeed, as will be shown in this essay, there is a deep and prevailing focus on mechanisms within epigenetic study, with seemingly less focus on what the mechanisms actually do at the organismal phenotypic level, especially across generations. As will be argued, epigenetics has a potentially profound role in our understanding of inheritance, natural selection and evolution and deserves more attention.

## 2. What Is “Epigenetics”?

The term “epigenetics” was coined by Waddington C.H. in his 1942 paper entitled “The Epigenotype” [1]. This was, of course, before DNA was discovered and before the complexity of gene expression was appreciated. Against this backdrop, the field of epigenetics at first slowly developed, gaining speed in recent years. For an introduction to the plethora of both reviews and research papers on the topic, see [2,3,4,5,6,7,8,9]. Since Waddington penned his 1942 article, many researchers have attempted, to varying degrees of success, to define, redefine or simply explain the term epigenetics. Indeed, the NIH PubMed database (see more on this below) includes well in excess of 300 papers that contain the phrases “definition of epigenetics” and/or “define epigenetics” and, so, are presumably in some way weighing in on defining epigenetic mechanisms and phenotypes. Authors persist in offering up their own definition of what comprises “epigenetics” and the criteria for scoring epigenetic phenomena; an appreciation for this complex semantic landscape can be gleaned from [2,7,10,11,12,13,14,15,16,17,18,19]. Unfortunately, progress in epigenetic research is being hampered by a lack of clarity as to what is epigenetics. As Deans and Maggert [11] have so aptly summed up, the current epigenetic semantics is as follows:

“Some employ epigenetics to explain changes in gene expression, others use it to refer to transgenerational effects and/or inherited expression states. This disagreement on a clear definition has made communication difficult, synthesis of epigenetic research across fields nearly impossible, and has in many ways biased methodologies and interpretations.”

The great science philosopher Karl Popper opined that “…..*we should altogether avoid, like the plague, discussing the meaning of words*.”[20] With all due respect to Popper, and to underscore the concern expressed by Deans and Maggert [11], those of us interested in epigenetics do have the responsibility of coming together around a common set of comprehensive definitions, as complex as necessary and as simple as possible, of epigenetic phenomena. Having made this observation (and likely to the relief of the reader aware of the semantic confusion in the definition of epigenetics and epigenetic phenomena), it is not the purpose of this article to attempt to enforce either a new or an existing favored semantic framework on a vibrant yet often chaotic field. To this author, at least, *all* truly epigenetic phenomena are grist to the mill as we sort out the role of epigenetics in inheritance, natural section and evolution, though some phenomena have far more relevance to evolutionary biology than others.

This essay, then, will skirt the issue of specific definitions of epigenetics and will employ a simple (perhaps simplistic) conceptual framework that differentiates between the use of “epigenetics” to describe intragenerational phenomena and its use to describe transgenerational phenomena. This straightforward dichotomy allows us to leave somewhat in the background the voluminous literature on the intragenerational changes in gene expression within an individual during its lifetime and to focus more acutely on the inheritance of characteristics. Consequently, for the purposes of the rest of this discussion, consider the following division:

Intragenerational epigenetics: modification of gene expression through epigenetic marks (e.g., DNA methylation, covalent histone modification, microRNA action) that results in a modified phenotype, often considered at the molecular/cellular level, within an individual’s lifespan. Those who focus on this aspect of epigenetics will be familiar with phrases, such as “…. the epigenetics of cancer…” or “…. the epigenetics of cardiovascular disease”.

Transgenerational epigenetics: the inheritance of a modified phenotype from the parental generation without changes in genes or gene sequence. The same epigenetic markers mentioned above may be responsible, but a focus in this category is on the act of *inheritance.* Note that a few authors have used the more restrictive term “cross-generational” to identify inherited traits resulting from the direct exposure of gametes of the F*_1_* and F*_2_* generations while within the body of the P*_0_* generation, as well as maternal effects, such as provisioning [2,21,22,23,24,25]. While from a mechanistic point of view, it is quite important to distinguish these different forms of epigenetic inheritance, this essay simply uses the Latin root “trans” in creating the broad category of “transgenerational” epigenetics, without attempting to link the inheritance to a specific mechanism. This may disappoint more mechanistically-focused researchers in epigenetics, but at this immature stage of exploring the role of transgenerational epigenetics in evolution and evolutionary process, the phenomena themselves may actually be as important as the specific mechanisms. Finally, it is important to point out that these two foci or categories of epigenetics (I will not call them “definitions”)—intragenerational and transgenerational—are not mutually exclusive. A change in gene expression and, thus, in phenotype in the adult P*_0_* generation caused by DNA methylation, for example, can also be carried over into the F*_1_* generation or beyond.

One might reasonably ask, what is the primary focus of workers in the field of epigenetics: intragenerational or transgenerational? This is not an easy question to answer, and even a thorough reading of a few select papers on the topic with their carefully chosen references is unlikely to provide further illumination. Thus, a survey of the literature was performed to determine the relative emphases on the two broad foci of epigenetics outlined above before moving on to explore the role of epigenetics in evolutionary biology.

## 3. A Survey of Epigenetic Research Papers: Methodology

As indicated above, various researchers use the word “epigenetics” and its derivatives in a wide variety of ways. The use of the word “epigenetic(s)” was probed by analyzing published papers in the PubMed database. Maintained by the National Center for Biotechnology Information of the U.S. National Institutes of Health (http://www.ncbi.nlm.nih.gov/pubmed/), this database catalogues >25,000,000 journal articles in the life sciences, broadly defined. Incidentally, this represents nearly half of all journal articles in all fields estimated to have ever been published [26].

In the current survey, searches were conducted to determine the total number of papers that contained carefully selected key search terms. The invariant, primary search term was “epigenetic”, which identified articles whose title or abstract contained the noun “epigenetics”, as well as the adjective “epigenetic”. Papers within this category are hereafter referred to as “epigenetic papers”. The variable secondary search term was based on a more specific probe of the epigenetic papers. Searches were conducted using a Boolean search methodology to assemble the total number of papers containing, for example, the keyword “physiology”. Secondary search terms also considered related adjectives, e.g., “physiological”, to minimize the creation of duplicate entries. Words with broad, multiple meanings, e.g., “generation”, were avoided because of the potential dilution of the search with papers that used non-pertinent meanings of a word (e.g., “generation” as in creation, as opposed to the description of offspring and parents: P*_0_*, F*_1_*). Secondary search terms were grouped around common themes. Thus, for example, the general category of “epigenetics” + “disease” actually included secondary search terms of not only “disease”, but also “illness”, “death”, “pathology”, “cancer”, “clinic”, “hospital”, *etc.*

There are multiple caveats to this survey, some obvious, some subtle, that need to be laid out before considering the results.

1. Searches of the PubMed database only identified papers whose title or abstract contained a search term; that is, the body of the paper was not part of the search. Thus, it is possible that papers discussing epigenetics in some form or other have been overlooked. However, in a sense, this eliminated trivial references to epigenetics and focused the survey on those papers that actually thought epigenetics was important enough to include in the abstract, if not the title itself.
2. The choice of search terms reflects the author’s view of biology. Thus, for example, “evo-devo” may arguably not be a “discipline” within the life sciences, *per se*, but it does reflect an actively growing area of biology.
3. This survey were carried out in February of 2016. The PubMed database is expanded daily, if not hourly. Indeed, estimates are that an average of ~1 paper a minute is added to this database, but in batches. Therefore, this survey is unique to that specific month and year and in this sense is not exactly replicable by the reader using the current on-line database. However, the incremental changes are unlikely to affect the outcomes of the survey or its conclusions, at least in the short term, given PubMed’s huge article database.
4. Importantly, any survey is affected by the source of its data, and this one is no different. While PubMed contains an enormous number of scientific articles, as implied by its name, the database does primarily focus on biomedical research. Certainly, there are journals in the area of evolution and ecology that are not indexed by PubMed (though plant biology, for example, generally is well represented). Thus, there may be a bias towards underrepresentation of evolution papers in this database. On the other hand, as the survey goes on to reveal, the near complete absence of evolutionary papers mentioning epigenetics compared to the overall number of epigenetic papers speaks volumes about the failure to link epigenetics with biology, when considering the many life science journals in animal, plant and microbial science that are indexed by PubMed.
5. No distinction was made between primary research papers and review papers in this survey.
6. Collectively, the caveats in this survey do point out some sources of inaccuracy in the survey. For example, the secondary search terms are somewhat subjective choices made by the author. Moreover, in some limited instances, papers are undoubtedly double counted, e.g., a paper containing the words “epigenetic”, “illness” and “death” contributes to the paper count for the overall category of “disease”. Important to emphasize is that the goal of this survey was simply to seek out general patterns, not to provide an accurate statistical analysis that would characterize a true meta-analysis. However, given the enormous number of papers in the PubMed data base, nearly 50,000 of which reference epigenetics in some form or other, it is assumed that meta-patterns will emerge despite these inherent limitations of the analytical approach. To emphasize the general nature of these findings, data on percentages are reported to the nearest whole number (but even rounding to the nearest 10% would not obscure the emergent patterns, as is evident below).

Now, having considered the caveats and limitation to the survey, what does it reveal?

## 4. The Reach of Epigenetic Research in the Life Sciences

### 4.1. Intragenerational and Transgenerational Epigenetics

**Figure 1:**
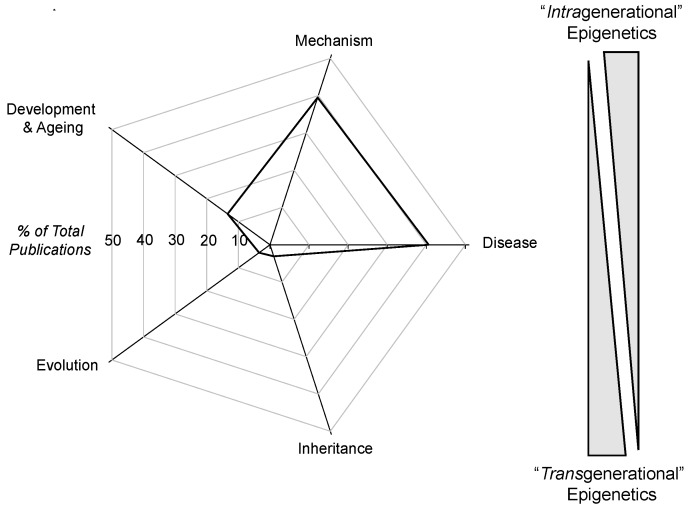
Radar diagram showing the relative distribution of publications drawn from the PubMed database (http://www.ncbi.nlm.nih.gov/pubmed/) that contain the search terms “epigenetic(s)” and one of five focus areas. The graphic to the right indicates a gradient between intragenerational and transgenerational epigenetics based on the percentage of epigenetic papers emerging from each area of study indicated in the radar diagram. Thus, epigenetic papers with the terms “development and ageing” or “disease” are assumed to be more likely to be addressing intragenerational issues, such as evolution, while epigenetic papers mentioning “evolution” or “inheritance” are viewed as more likely to be focusing on transgenerational epigenetic events. See the text for an additional discussion.

The first phase of the survey determined the relative frequency of the occurrence of intragenerational *vs.* transgenerational epigenetic papers. The actual use of these two specific terms as the secondary search term was not productive: only slightly above 1% of all epigenetic papers actually contained either or both of these adjectives. Subsequently, five major categories of epigenetic papers were formed for this survey. Epigenetic papers including the terms “mechanism”, “disease” and “development and ageing” (and their related topics) were considered to be more representative of an intragenerational perspective, while epigenetic studies, including the terms “evolution” and “inheritance”, were considered to be more representative of epigenetic papers with a transgenerational component to them (however little that might be) Of course, there are transgenerational epigenetic papers that discuss the mechanism of inheritance, and these would be represented in both the “mechanism” and “evolution” category. However, as is evident from Figure 1, the majority of the focus of epigenetic studies was on mechanism and disease states in approximately equal measure. Indeed, <7% of papers referencing epigenetics also mentioned either evolution and/or inheritance. Noteworthy is that while transgenerational epigenetics studies have revealed many instances of epigenetic inheritance of disease/pathologies (e.g., [3,27,28]), the epigenetic inheritance of mal-adaptive modified phenotypes receives little attention compared to the “here and now” of diseases that develop in an individual’s life span. These findings are not surprising, as even a quick examination of a sample of papers comprising the epigenetic literature reveals intensive discussion of *mechanisms* of epigenetic phenomena, especially as they relate to human health and disease. Additionally, to no one’s surprise, funding follows disease and its prevention and cure, which has greatly enabled the growth of epigenetic studies.

### 4.2. Epigenetics and Taxon

**Figure 2:**
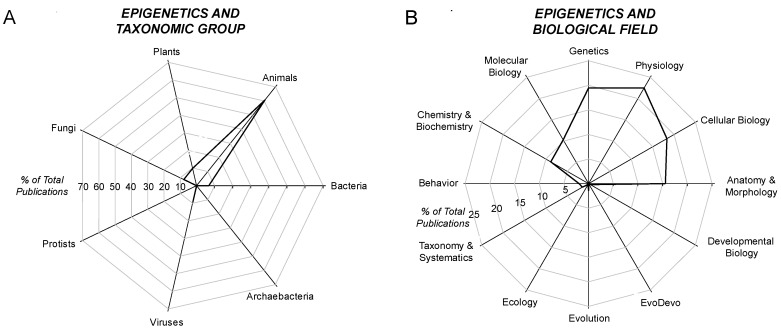
Radar diagram showing the relative distribution of publications on epigenetics drawn from the PubMed database (http://www.ncbi.nlm.nih.gov/pubmed/). (**A**) distribution of publications that contain the search terms “epigenetic(s)” and one of seven biological taxa; (**B**) distribution of publications that contain the search terms “epigenetic(s)” and one of 12 biological fields.

The survey next explored the taxonomic distribution of epigenetic papers using the secondary search terms (and their adjectives) of “animals”, “plants”, “fungi”, “protists”, “bacteria”, “archaebacteria” and “viruses” [29]. Approximately 60% of epigenetic papers contained the search term “animal(s)”; ~10% contained “plant(s)”; and near negligible numbers of epigenetic papers specifically mentioned any of the other major taxa (Figure 2A).

### 4.3. Epigenetics and the Biological Field 

The survey next considered epigenetic papers that included one of 12 major biological fields (Figure 2B). The vast majority (~95%) of epigenetic papers that even mentioned, if not actually discussed, a particular biological field was clustered in just six areas: chemistry/biochemistry, molecular biology, genetics, physiology, cellular biology or anatomy/morphology. Occurring at a very low frequency in the epigenetic literature were the fields of behavior (~2% of papers), taxonomy/systematics (1%–2%), evolution (a little above 1%) and, all being less than 0.5% of the epigenetic papers, development, ecology and evo-devo.

Combing the survey on biological fields and taxa reveals how some areas of epigenetics are almost completely unexplored. For example, combining “epigenetics” + “plant” + “evo-devo” yielded only two papers among the ~50,000 epigenetics papers warehoused in PubMed. Similarly, “epigenetics” + “virus” + “ecology” yielded just three papers. Yet, as we will now turn to, the role of epigenetics in the biology of all of these taxa may be profound.

## 5. Investigating Epigenetic Inheritance: The Challenges

Epigenetic papers characterized as predominantly focusing on transgenerational issues (see Section 4.1 above) account for only 5%–7% of all epigenetic papers, so it comes as no surprise that a similarly small proportion of epigenetic papers revealed in this analysis specifically consider transgenerational inheritance of a modified phenotype. Why is there so little explicit focus on epigenetic inheritance, as opposed to intragenerational epigenetics, typically associated with disease states? In partial answer to this question, and speaking pragmatically, there are numerous hurdles to well-conducted studies of transgenerational epigenetic inheritance, as will now be explored.

### 5.1. The Resource Cost of Epigenetic Studies

It is relatively easy to do thought experiments in epigenetic inheritance, but quite another thing to carry out actual experiments, for several reasons. First, there is an extended investment of time not found in intragenerational studies; put simply, one does not leap to study epigenetic inheritance in elephants, with their life span of up to 70 years. The study of transgenerational epigenetic inheritance by definition requires the maintenance and breeding of more than one generation of organisms and the significant costs in time, money, space, *etc.*, that are required (even for microorganisms). Not surprisingly, then, studies of basic questions in epigenetic inheritance often turn to include animal (*Drosophila*, *Caenorhabditis*) and plant (*Arabidopsis*) models with relatively short generation times (e.g., [30,31,32,33,34,35,36,37,38,39]).

### 5.2. Elusive Epigenetic Mechanisms

A second factor that may complicate the study of epigenetic inheritance is difficulty in teasing out underlying mechanisms (which, as is evident from Figure 1, is a major driver of epigenetic research). Some researchers differentiate epigenetic inheritance due to parental effects or provisioning of eggs with hormones and other substances (so-called context-dependent epigenetic inheritance, which in some cases can span a few generations) from epigenetic inheritance due to the experiences of the egg cells and/or sperm cells and/or stem cells that produce them (so-called “germ-line dependent” epigenetic inheritance). The latter can span F*_1_* and F*_2_* generations at most [2,25,27]. Indeed, some researchers go to great lengths to argue what is and is not epigenetic inheritance, but likely, Waddington C.H. would not be impressed. In the end, both types of epigenetic transfer result in a modified phenotype in the F*_1_* generation. The relevance of the mechanism (and thus, the effort to identify it) depends in part on the nature of the questions being asked. If one asks “How do I prevent this epigenetic disease?”, then knowing the mechanism is all-important. If, however, one asks “Does epigenetic inheritance drive the evolution of a population or species?”, the actual mechanism may or may not be pivotal to the answer.

### 5.3. The Complexities of Epigenetic “Dynamics”

A third reason why studies of transgenerational inheritance are difficult to conduct and interpret is that it increasingly appears that a complex, yet often subtle or even hidden, set of transgenerational epigenetic “dynamics” can prevail [40,41]. Essentially, most researchers in the field appear to consider an epigenetically-inherited trait as digital: it is either present/on or absent/off. Thus, experiments are typically conducted where the P*_0_* generation is subjected to some form of stressor, and the F*_1_* generation (in fact, often just the *neonates* of the F*_1_* generation) are scored as to whether they have the trait or lack it. Yet, evidence is accruing that epigenetically-inherited traits in some organisms may take a few generations to actually “wash in”, even being completely absent in the initial F*_1_* generation [42]. Similarly, traits may “wash out” over several generations [42]. Overall, however, the assessment of the presence and extent of the epigenetic inheritance of a trait may well pivot on whether multiple generations are observed and on the threshold level for the detection of a trait within each generation [41]. Interestingly, the trait of reduced body mass in the F*_1_* generations resulting from parental P*_0_* hypoxia exposure dissipates (washes out) both over time as individuals grow, but also washes out over the course of the three successive F*_1_* broods produced by the mother [31], though the complexities introduced by germline exposure (Section 5.2) require further experimentation to clearly identify what mechanism is at play.

Epigenetic dynamics not only is evident across multiple generations, but also across multiple broods *within* generations. For example, exposure of adult *Daphnia magna* to hypoxia results in the birth of offspring with initially reduced body mass. However, this effect is only evident for the first 3–5 days after birth and only occurs in the first two broods of the mother, being absent in her third brood [31]. Thus, this epigenetically-induced phenotype of reduced body mass “washes out” over the course of development in the F*_1_* generation and over multiple reproductive events in the P*_0_* mother. Some researchers in epigenetic inheritance are seemingly unaware of these dynamics and fail to incorporate them into their experimental design. This may account for many “false negatives” and the underreporting of transgenerational epigenetic inheritance.

### 5.4. Keep Calm and Carry on with Transgenerational Epigenetic Research

As is evident from the above assessment, epigenetic transgenerational research is neither easy nor straightforward. Yet, the obstacles are not insurmountable. Tractable animal models with short generation times are available. These same models can be used in experimental designs of sufficient complexity to delineate mechanisms. Experimental designs can also be tweaked to reveal the epigenetic dynamics of the wash in/out of epigenetically-inherited traits.

## 6. Epigenetics in a Dynamic Environment: Consequences of Rapid and Widely-Distributed Phenotype Switching

### 6.1. Epigenetically-Inherited Phenotypes: Neutral, Advantageous or Disadvantageous?

The consequences of an epigenetically-inherited phenotype depend in large part on what the effect of that phenotype is on the overall fitness of the individual bearing it. Just like genetically-inherited phenotypes, epigenetically-inherited phenotypes can be neutral, advantageous or disadvantageous. In the field of medicine, most focus is on disadvantageous epigenetically-inherited phenotypes that can lead to disease states. Advantageous epigenetically-inherited phenotypes have received less attention in human health, yet certainly exist [43]. Indeed, if they can be controlled and managed, advantageous phenotypes arising by transgenerational epigenetic inheritance may have potentially large impacts on medicine [44,45] and on agriculture [10,46,47,48,49,50]. To highlight a concrete example of beneficial epigenetic inheritance, consider the transgenerational epigenetic inheritance of hypoxia resistance in zebrafish [7]. As a result of parental (P*_0_*) exposure to 2, 3 or 4 weeks of hypoxia (15%), F*_1_* larvae had greater hypoxic resistance than controls whose parents had not experienced hypoxia. Importantly, it was not just an individual larvae or two that were hypoxic resistant, but rather statistically, the entire F*_1_* population had elevated hypoxic resistance. Similarly, we have recently observed that F*_1_* of zebrafish parents exposed to polycyclic aromatic hydrocarbons (PAHs) showed enhanced resistance to these toxicants when compared to control larvae whose parents were not exposed (Martinez-Bautista N. and Burggren, W. unpublished data [51]). Again, most of the population, not just a few individuals, had resistance greater than the controls whose parents were not exposed. These two examples highlight that more experimentation of potentially advantageous epigenetically-inherited phenotypes is warranted. 

### 6.2. Comparing the Time Courses of Genetic and Epigenetic Inheritance

One of the basic tenets of evolution is that natural selection shapes populations and species over evolutionary time. Natural selection acts on organisms with enhanced or diminished fitness, derived from the accumulation of mutations. The resulting phenotypic modifications are enhanced (or not) by these mutations, but the phenotypic switch at the population level and beyond typically occurs over hundreds or thousands of generations as the genotype leading to a modified phenotype of greater fitness slowly inserts itself into the general population or, alternatively, a genotype leading to lesser fitness is eliminated from the population [52]. Klironomos *et al.* [53] have provided a simple, but informative model of how increases in fitness in a population can derive from either epigenetic or genetic changes in a population over tens of thousands of generations. However, the effect of epigenetic inheritance may not only be potentially broad and sweeping, but may also be felt immediately in a population [52,53,54,55,56]. To underscore this point, consider a phenotype that is *advantageous* in an environment when a specific stressor that occurs intermittently. Unlike an advantageous gene mutation that affects an individual and then, perhaps, spreads slowly through the population and beyond over many generations, epigenetic inheritance can simultaneously affect many (if not most or all) of a single generation of an entire population. Why? While there is certainly some variation in epigenetic markers between individuals in a population (see below), whether they result in an advantageous or disadvantageous phenotype, epigenetic markers will arise in response to an environmental stressor far more broadly and quickly within a single generation of a population than will a single point mutation occurring in a single individual. Assuming that all individuals in a population of a species presumably experience an environmental stressor at the same time and to a similar extent and that many of the individuals in that population will as a consequence possess the same epigenetic markers, then an epigenetically-switched phenotype should affect many if not most individuals in the population.

**Figure 3:**
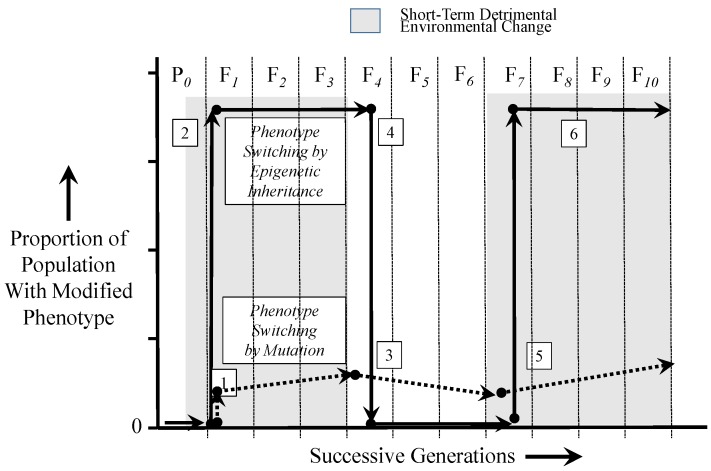
A comparison of phenotype switching in a population occurring by inherited point mutation *vs.* inheritance through the effects of epigenetic markers. Events 1, 3 and 5 indicate proportional changes in a hypothetical population resulting from phenotype switching by point mutation that are advantageous during environmental stress, but otherwise disadvantageous (or at least energetically costly). Events 2, 4 and 6 indicate proportional changes in the population resulting from epigenetic phenotype switching. See the text for an additional explanation.

The scenario described above is depicted in Figure 3, which compares changes in a population of individuals with an advantageous phenotype arising by either epigenetic inheritance or by mutation. This scenario assumes firstly that the switched phenotype (either from genetic or epigenetic inheritance) is advantageous only in the presence of a deleterious environment, which persists over several generations (specifically, four generations in this scenario) before returning to normal, favorable environmental conditions. Second, this scenario assumes that upon return to the previous normal environment, the newly-switched phenotype is now disadvantageous and possibly lethal. Third, this scenario revolves around only a simple point mutation and, thus, ignores the complexities of pleiotropy, including antagonistic pleiotropy. Fourth, the scenario assumes that an epigenetically-inherited phenotype may persist over more than one generation. Indeed, abundant evidence now exists of epigenetically-inherited phenotypes persisting over multiple generations (e.g., [6,15,42,57,58,59]) before either suddenly disappearing or more slowly “washing out” [41].

As Figure 3 illustrates, a mutation may result in an advantageous phenotype in only a single individual in a population (Event 1). Advantageous mutations occur at low frequency, become difficult to establish in the population and additionally may be easily lost to genetic drift [60,61]. Thus, this advantageous mutation (Event 1) is only slowly amplified by natural selection over numerous generations, at best. In contrast, an epigenetic phenotypic switch brought on by a deleterious environment can immediately aid in the survival of a potentially large proportion of a population (Event 2), since even allowing for the heterogeneity of epigenetic markers in a population, many in that population may have the epigenetic markers resulting in the modified phenotype. With dissipation of the deleterious environment, however, the individual(s) with the original mutation must cope with the newly disadvantageous phenotype, which cannot be eliminated from the gene pool, except by death of the individual or an unlikely second mutation back to the original gene form (Event 3). In contrast, however, the epigenetically-switched phenotype, now newly disadvantageous in the face of the return to the original environmental condition, is immediately lost by reversion to the original phenotype (Event 4). With a return of the deleterious environment after several generations, the mutant genotype and its phenotype (if they even survive the intervening return to the previous normal environment) will increase only slowly once again in the population at a rate enabled by natural selection (Event 5). Again in contrast, the epigenetically-inherited advantageous phenotype can result in the rapid re-appearance of the advantageous switched phenotype appearing in a large proportion of a population’s individuals (Event 6).

Important to acknowledge is that the scenario depicted in Figure 3 takes an “either-or” approach for epigenetic or genetic inheritance. That is, that populations are shown in this figure to either persist by epigenetic inheritance or by genetic inheritance of an advantageous phenotype, but not necessarily both. We know this approach to be an oversimplification, because presumably, there are also genetic changes that occur in populations changing by epigenetic modification. In fact, it is difficult to separate out such simultaneous phenotypic changes caused by this duality [25,53,57,62,63,64].

## 7. An “Epigenetic Advantage” in Changing Environments

### 7.1. Epigenetics in a Temporally-Complex Environment

The epigenetically-enabled survival described above and illustrated in Figure 3 becomes all the more relevant in non-stable, non-equilibrium environments subject to frequent, major changes; e.g., [5,52,53,65,66,67,68,69]. Epigenetic modifications are increasingly being viewed as providing a rapid change in phenotype that simply cannot occur quickly enough through genetic mutation [52,53,54,55,56,70,71,72,73]. Such epigenetic patterns and the phenotypes they have affected can be inherited over multiple generations, as alluded to earlier.

Of key importance is the fact that a phenotypic switch occurring broadly throughout a population caused by epigenetic inheritance is rapidly reversible (Figure 3). This differs from a mutation that requires an unlikely reversion to the pre-mutation genotype. Importantly, these characteristics of transgenerational epigenetic inheritance can allow the survival of organisms with temporarily low fitness resulting from a temporary environment change population [40,74,75,76]. Notably, however, the modified phenotype that was advantageous for ameliorating the effects of the stressor may be at best of no benefit to the individuals in the population; rather, this phenotype may actually be disadvantageous once the stressor wanes or disappears. Phenotype switching arising from mutation thus leaves surviving individuals “stuck” with that modified phenotype, which may now be, for example, more energetically expensive to maintain, after the environmental stressor disappears [40]. In stark contrast, an epigenetically-inherited phenotype may only persist as long as the environmental stressor is evident, but then, the modified phenotype (and its possible energetic costs) are “sunsetted” as the environmental stressor disappears. Consider a hypothetical population of freshwater fishes in a small, drought-plagued pond encountering severe hypoxia over several generations. A mutation resulting in thinner gill membranes might assist oxygen loading at the gills in hypoxic conditions. These thinner gills also may result in more energetically expensive osmotic uptake of water, but it is a reasonable trade-off, since the higher cost of osmoregulation is offset by the fact that the fish can continue to acquire the oxygen needed to survive in a hypoxic environment. However, when rains return, water levels rise, and water returns to full oxygen saturation; these thinner gills now carry a critical disadvantage (enhanced cost of osmoregulation) with no additional advantage to gas exchange when in well-aerated water. In this situation, fishes epigenetically inheriting thinner gills as a result of parental hypoxic exposure (presumably a high proportion of the population) revert to the original phenotype (thicker gills with reduced osmotic water uptake) when normoxic conditions return (in practice, few examples exist in fishes of transgenerational epigenetic inheritance of advantageous modifications at the level of organ structure or function, but epigenetic inheritance of hypoxia tolerance has been demonstrated in the zebrafish as described above [77]).

**Figure 4:**
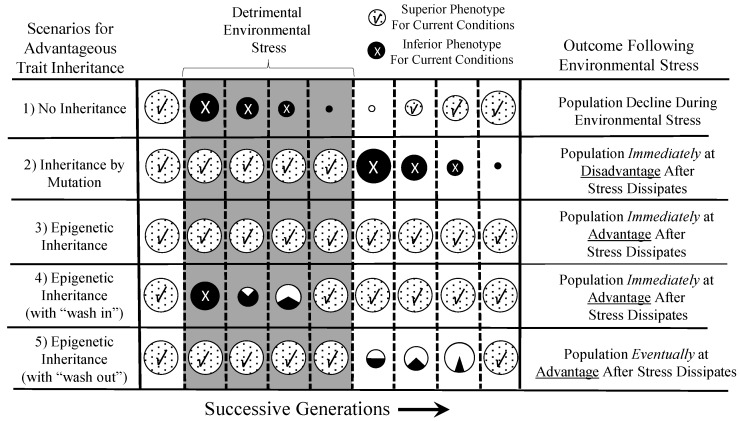
The “epigenetic advantage” of epigenetic inheritance in a population living in an environment with periods of detrimental conditions. Each circle represents a population in transition over many generations, with the size of the circle reflecting the size of the population. All populations are assumed to have an advantageous phenotype in the non-detrimental environment at the beginning of the graph on the left. Upon the appearance of a critical environmental stressor, successive generations of each population either thrive with a phenotype, acquired by mutation or epigenetic inheritance, or they go into decline. Similarly, as the detrimental environment stressor wanes or disappears, the subsequent generations either have a phenotype advantageous or disadvantageous with respect to the original stressor-free environment. See the text for an additional explanation and discussion.

The “epigenetic advantage” in a seasonally- or annually-dynamic environment where environmentally-related stressors may last only a few generations is graphically expanded upon in Figure 4, through several specific scenarios. In Scenario 1, a species facing a detrimental environment over several generations may fail to thrive if no advantageous modification of phenotype occurs. Indeed, the environmental stressor could take on the trappings of an extinction event. However, in Scenario 2, a population could survive, if not thrive, through the occurrence in an individual or individuals in that population of a spontaneously-arising mutation that is advantageous in the face of the new environmental stressor. However, when the environmental stressor disappears, the population is “stuck” with the phenotypic modification, which may be disadvantageous or, at least, costly. In contrast, Scenario 3 depicts how an epigenetically-inherited advantageous phenotype can be important to the survival of the population during the period of environmental stress, but can “sunset” when the stressor disappears. This epigenetic inheritance allows the population to revert back to the original phenotype that was advantageous in the original environment. Scenario 4 and 5 are versions on this theme, showing how transient (in evolutionary terms) advantageous phenotypes acquired by epigenetic inheritance can “wash in” (4) or “wash out” (5), essentially reflecting a lag-time to epigenetic inheritance across generations.

Table 1 summarizes, compares and contrasts the characteristics and effects of gene mutation and transgenerational epigenetic inheritance. Essentially, the epigenetic advantage in a dynamic environment derives from the immediate effect potentially in much of a population [52], the potential for an advantageous phenotype to help “bridge” a period of general low fitness by the population [40,74,75,76] and the ability for the switched phenotype to be “sunsetted” when the environmental stressor dissipates [2].

### 7.2. Epigenetics in a Spatially-Complex Environment and the Exploitation of New Ecological Niches

Epigenetic transgenerational inheritance can not only be advantageous in a temporal sense, as discussed in Section 7.1, but additionally may assist spatial expansion of a species into new niches. Niche theory indicates that a given species will persist in its niche as a result of the cumulative impact of all of the factors that affect its persistence [78], and this should correctly include the potential for epigenetic phenotype switching. For example, environmentally-induced DNA methylation polymorphisms allow flower-dwelling yeasts to exploit a much broader range of niches than would otherwise be the case [78,79]. Northward range extensions in the common dandelion (*Taraxacum officinale*) are similarly linked to between-population variations in DNA methylation [80]. While the above examples are sufficiently compelling to warrant the expansion of this line of research, to date, the interleaving of phenotypic plasticity, epigenetic inheritance and ecological theory has been scant, at best. To return to the survey, <1% of epigenetic papers mention ecology in the title or abstract. In summary, the emerging field of “ecological epigenetics” is still in its infancy (for an introduction, see [16,81,82,83,84]), but shows great promise in understanding niche exploitation in a spatially- (and temporally-) complex environment.

## 8. A Role for Epigenetics in Evolution

To this point, this essay has discussed substantial qualitative and quantitative differences that emerge between genetic and epigenetic inheritance of new phenotypes. Moreover, these differences —essentially the magnitude of the effect in a population and the rapidity of the onset and dissipation of the modified phenotype—persist even when taking into account substantial genetic and epigenetic variation within a population. Inheritance is necessary for evolution, but the phenomenon of inheritance does not constitute evolution by itself. We now turn to the consideration of what the consequences of epigenetic inheritance are, if any, for evolutionary processes.

### 8.1. Is Epigenetics Even Relevant to Evolution?

The survey of the PubMed database reveals that the word “epigenetic” appears in <1% of all indexed “evolution papers”, and <4% of all epigenetic papers contain the word “evolution” (Figure 2B). Why so little cross-reference? One answer is that researchers may have concluded that there is no role of epigenetic inheritance in evolutionary biology. Indeed, there is a significant group of researchers that view epigenetics as uninvolved, or at least unimportant, in the process of evolution. For an introduction to this debate, see [84,85,86,87,88,89]. Yet, a key question remains largely unanswered: “To what extent, if any, do epigenetically-inherited phenotypes subsequently become fixed in the genome?” Put differently, “Do epigenetic phenomena affect evolution or does epigenetic inheritance simply co-exist independently alongside evolutionary processes, having no influence on the genotypic mix of a population”? Such questions are pivotal in the arguments for the inclusion of epigenetic inheritance into the so-called modern synthesis in evolution (see Section 8.3).

Therefore, why not assume that epigenetically-inherited characters can be fixed in the genome? Objections to inserting epigenetics into the established evolutionary paradigm often revolve around reasonable concerns involving (1) the perceived lack of substantial and compelling experimental evidence of the permanent fixation of epigenetically-inherited traits into the genome, occurring broadly across taxa and (2) no clear delineation of mechanisms by which this might occur. Thus, most discussions of the role of epigenetics and evolution appear to be based on an abundance of theory and theoretical constructs and relatively little unambiguous data. While not all evolutionary biologists immediately (or might ever) endorse a role for epigenetics in evolution, the current author concludes on largely (but not entirely) theoretical grounds that epigenetics most certainly does influence the evolution of a population, at least indirectly, though not necessarily in the way that many in the debate might think, as will now be considered.

### 8.2. How Epigenetic Inheritance Influences Evolution

#### 8.2.1. Beyond the Modern Evolutionary Synthesis

Many evolutionary biologists have embraced the notion that epigenetics plays a role in evolution, while many have yet to make that conclusion. Consequently, a swirling evolutionary discourse (in fact, a controversy) has developed about modern evolutionary theory and the need for new, extended or rehashed evolutionary synthesis. A key part of that discourse is how transgenerational epigenetics factors into inheritance and evolution, with emerging concepts and terms, such as “soft inheritance”, “soft genome”, “cell memory”, “epigenetic marks” and “cultural transmission” being inserted into more traditional evolutionary theory, as represented by the modern synthesis [18,90,91,92,93,94,95,96,97,98,99,100,101,102,103,104,105,106,107,108,109].

An in-depth immersion in the as-yet-unresolved debate about the role of epigenetics in shaping evolutionary theory is beyond the scope of this essay. Noteworthy in passing is that, perhaps because of the classic biological training paradigm that closely links inheritance with evolution, there is a tendency by some to assume that evolution has occurred, or is occurring, if a phenotype apparently becomes “fixed” in the population, as ascertained by its continual appearance over several consecutive generations. This simple litmus test for evolution can erroneously lead to the conclusion that an epigenetically-inherited phenotype has become incorporated into the genome when, in fact, the persistence of an epigenetically-inherited phenotype can result from continued inheritance of epigenetic markers over numerous generations. Consider the zebrafish exposed to polycyclic aromatic hydrocarbons, such as benzo-a-pyrene. The morphological effects (pericardial edema) may not disappear until the F*_3_* of F*_4_* generation (and may not even appear in the F*_1_* generation) [42]. In a few more extreme, yet illustrative examples, epigenetically-inherited phenotypes caused by RNA interference persist across as many as 50 generations in *Caenorhabditis elegans* [34,58,110,111]. In the plant toadflax, epigenetically-inherited phenotypes are said to persist across possibly hundreds of generations [59]. Thus, the mere persistence of a modified phenotype across as many generations as most investigators have the patience to wait for is in and of itself insufficient evidence for the fixation of epigenetically-modified traits into the genome. Yet, there is evidence and theoretical arguments for both the direct and indirect involvement of epigenetics altering the genome, as will now be considered.

#### 8.2.2. Direct Incorporation of an Epigenetically-Inherited Phenotype into the Genome

Numerous papers have been published over the last several decades that have analyzed how genomic DNA methylation has changed (or remained stable) over evolutionary time scales. Typically, these studies reveal the functional specificity of species-specific DNA methylation, correlate of evolutionary DNA methylation signatures, document extensive phylogenetic conservation of DNA methylation mechanisms and show paramutation through RNA interference. The phenomenon of paramutation—where directed allelic interactions create heritable changes in the state of an allele—is being intensively studied in plants [50,112,113] for its implications to crop productivity, for example.

Collectively, then, compelling evidence points to epigenetic influences on speciation with both short- and long-term evolutionary implications [19,50,54,112,114,115,116,117,118]. The fascinating topic of the influence of epigenetics in speciation is beyond the scope of this essay; for a comprehensive review of that literature, see [107].

Confounding the determination of the role of epigenetics in evolution and speciation is the key question of whether epigenetically-acquired phenotypic traits themselves can actually become fixed intact, *i.e.* permanently added to the genome, as opposed to having the markers that generate them have indirect influence on the subsequent genome through genome destabilization, mutation, natural selection or other mechanisms. Mechanisms by which this might occur are emerging, but evidence remains somewhat circumstantial [4,19,27,119]. A considerable amount of attention is focused on methylated CpG islands, which are considered mutational “hotspots”, because the methylated cytosine can undergo spontaneous conversion to thymine, while methylated guanine converts to uracil in these islands. These mutations, not necessarily recognized as damaged DNA, are not subject to excision or correction by DNA repair mechanisms [19,120]. Consequently, the mutations become fixed in the genome. While interspecific differences in amino acid sequence have been attributed to CpG hypermutability in primates [121,122,123], it remains unclear as yet, however, whether CpG mutation is actually linked to the creation of phenotypes that actually alter fitness and, so, contribute to evolution and speciation.

#### 8.2.3. Indirect Epigenetic Effects on Evolution: Altered Gene Stability and Mutation Rates

Beyond the direct incorporation of an epigenetically-inherited phenotype into the genome lie multiple indirect effects of environmentally-induced epigenetic markers upon gene stability and mutation rates. Elevated levels of cytosine methylation increase mutation frequencies as a result of elevated levels of cytosine-to-thymine transitions and other DNA characteristics [4,19,106,107,124,125,126]. Additionally, DNA methylation can interfere with both DNA damage repair genes [127,128] and apoptotic pathways that help maintain gene integrity [129]. While these last two phenomena have been examined largely in the intragenerational context of human disease, they do lead to permanent genetic change. As such, these changes along with altered mutation rates from cytosine methylation dynamics can all contribute to evolutionary changes through their indirect effects on gene stability.

Important to emphasize is that these epigenetic effects on evolution are categorized as indirect because the action of the epigenetic markers generated by environmental stressors is not necessarily related (and most likely unrelated) to the actual phenotype that the markers created.

#### 8.2.4. Indirect Epigenetic Effects on Evolution: Altered Natural Selection

A second indirect effect of epigenetic inheritance is the creation of a phenotype that leads to differential natural selection of genotypes [6,53,54,56,114,115,130,131,132]. Such a process is, of course, evolutionary in nature and leads to permanent shifts in the genetic makeup of a population. A classic example of this is the so-called “Baldwin effect”, in which an animal’s ability to learn new behaviors (which we know can be epigenetically inherited) may affect its success in reproducing (e.g., [19,133].

To further develop the notion of indirect influences on evolution by epigenetically-driven natural selection, consider the following “knowns”:

(1) *Phenotypes can be epigenetically inherited.* Abundant evidence of epigenetic inheritance exists in numerous organisms, as already discussed.
(2) *Epigenetically-inherited phenotypes can be neutral, adaptive or maladaptive.* Again, this has been discussed above in the context of epigenetically-inherited disease, for example.
(3) *Variation in genotype exists between members of a population*. This is, of course, a basic tenet of Mendelian inheritance, although fitness is increasingly viewed as a complex combination of population density, genotype and genotype frequencies [98].
(4) *Variation in epigenetic markers exists between members of a population.* There is increasing appreciation that there is variation between individuals within a population (and in an individual as a function of age) in the DNA methylation or post-translational acetylation, methylation, phosphorylation or ubiquitination of histones associated with the nucleosome cores and the phenotypes they ultimately produce; e.g., [53,55,64,78,81,106,125,132,134,135,136,137,138,139,140,141,142,143,144]. There are also spontaneous changes in DNA methylation patterns [89,145]. Thus, both the epigenome and genome of a population show heterogeneity to a greater or lesser extent.

Perhaps most critically, consider that:

(5) *Within a population, a particular set of epigenetic markers may be distributed completely independently from a particular genotype.* That is, a particular genotype in a population may or may not be associated with a particular set of epigenetic markers and the associated phenotype.

**Figure 5:**
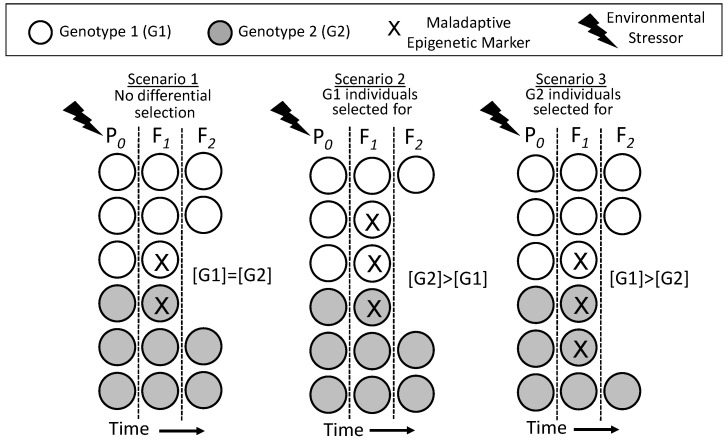
Changes in the distribution of genotype within a population as a result of differential selection against individuals with a maladaptive epigenetically-inherited phenotype (X) arising in the F*_1_* generation. An environmental stressor experienced by the P*_0_* generation induces maladaptive epigenetic markers in the F*_1_* generation, and the individuals with the maladaptive phenotype are selected against. Importantly, the distribution of epigenetic markers in the F*_1_* in Scenarios 2 and 3 is both variable and independent of genotype. See the text for an additional discussion.

If all points above hold, *then* epigenetics unequivocally affects evolution through its *indirect* action on the process of the natural selection of genotypes. Importantly, it is not required for the epigenetically-inherited phenotype to become fixed into the genotype to affect the evolution of traits. Rather, what drives the changing genotype of a population is the differential survival of individuals due to unevenly-distributed epigenetic markers creating advantageous or disadvantageous phenotypes that are subject to natural selection. Figure 5 illustrates this point. In Scenario 1, the parental generation is exposed to an environmental stressor, resulting in epigenetic inheritance of a maladaptive epigenetic marker (X) in the F*_1_* generation. The F*_1_* bearing this marker, which creates an inferior phenotype, happens in Scenario 1 to be evenly distributed between individuals of Genotype 1 (G1) and Genotype 2 (G2). Selection against the F*_1_* occurs evenly because of the presence of the epigenetic marker in both genotypes, so the proportion of the G1 and G2 genotypes is not changed across time in the population. In Scenario 2, more G1 individuals happen to have the disadvantageous epigenetic marker and its associated inferior phenotype than G1s. This results in a selective disadvantage for G1, which then decreases in the overall F*_2_* population as a result. In Scenario 3, the situation is reversed, with initially more G2 individuals bearing the disadvantageous epigenetic marker X. Consequently, G2 is selected against and declines in the overall F*_2_* population through natural selection on disadvantaged individuals.

### 8.3. Traditional Genetic Inheritance of Mechanisms for Epigenetic Inheritance

Inherited traits are often thought of as discrete structures, processes, behaviors, etc. Yet, the ability for phenotypic switching is also a heritable trait. For example, well documented is the inheritance of phenotypic plasticity, including developmental phenotypic plasticity and critical windows (e.g., [73,146,147,148,149]). What about the inheritance of an “epigenetic potential”? Somewhat ironically, especially given the continuing discourse involving the Modern and extended evolutionary syntheses discussed in Section 4, traditional genetics must be involved in the process of the creation and persistence in populations as a result of epigenetic inheritance. Indeed, several authors have opined that the mechanisms by which epigenetic inheritance and phenotypic switching occurs are themselves selected for and evolve over time [74,89,114,140,150,151,152]. A natural extension of this conjecture is that some populations of organisms may carry in their genes a lesser or greater ability to inherit a phenotype by epigenetic transgenerational transfer. Numerous studies have shown population differences in, for example, the degree of DNA methylation between populations of the same species in microbes [78,81], plants [136,153] and animals (including humans) [137,142,143]. While there is undoubtedly a stochastic component to such variation (e.g., [154,155,156]), variation in epigenetic markers between populations may be both evidence of and a mechanism for natural selection acting on the capability of organisms to show epigenetic phenotypic switching. How do these emerging phenomena play into a rethinking of the Hardy–Weinberg law, which states that allele and genotype frequencies remain constant in a population over generations in the absence of other evolutionary influences? Epigenetic studies on inheritance, natural selection and evolution and the phenomenon of paramutation collectively indicate that transgenerational epigenetic inheritance needs to be factored into the venerable Hardy–Weinberg law.

## 9. Conclusions and A Possible Future for Epigenetic Research

The epigenetic juggernaut is gaining momentum and is likely to touch most if not all aspects of biology in the coming years. While the importance of epigenetics in human disease is being intensively studied (especially intragenerational aspects), the role that epigenetics plays in inheritance and beyond to evolution is receiving much less attention, even though the implications may be far reaching. The importance of epigenetic inheritance in natural selection and evolution is not yet fully understood, and few definitive experimental studies have explored actual fitness enhancements over time in individuals and populations with epigenetically-inherited modified phenotypes, which by their nature are transient. Theoretical considerations, some of which have been explored in this essay, argue for the importance of thoroughly integrating epigenetic phenomena into the process of evolution. Whether this requires another theoretical synthesis is perhaps less important than aggressively moving forward to add actual experimental data to the few existing studies operating at the nexus of epigenetics, natural selection and evolution.

## Figures and Tables

**Table 1:** A comparison/contrast of the timing and reach of genetic and epigenetic inheritance.

Characteristic	Form of Inheritance	
	*Genetic*	*Epigenetic*
*Rapidity of Appearance in the Population*	Full onset in the F*_1_* generation	Onset in the F*_1_* generation, unless there is a “wash in” effect, in which case, full phenotypic switching may develop over multiple generations
*Numbers of Affected* F*_1_* *Individuals in the Population*	Typically one or at most a few F*_1_* individuals, with a slow increase in the allele (if an advantageous phenotype) or a decrease (if a deleterious phenotype) over a large number of generations	Many F*_1_* individuals receiving the same epigenetic markers
*Longevity in the Population*	Permanent, until the individual with the altered allele is eliminated by natural selection or the allele is modified by additional mutation	Transient, with continued epigenetic inheritance dependent on continued exposure to the environmental stressor, resulting in modified epigenetic markers (incorporation of epigenetically-inherited phenotypes into the epigenome is still being debated)
